# Examining the correlation between referee physical fitness, in-game performance metrics, and team dynamics in the Chinese super league

**DOI:** 10.1371/journal.pone.0318643

**Published:** 2025-05-28

**Authors:** Fei Zhou, Wei Zhang, Kongyun Huang, Yujie Jiang, Changjing Zhou

**Affiliations:** 1 School of Athletic Performance, Shanghai University of Sport, Shanghai, China; 2 School of Physical Education, Shanghai University of Sport, Shanghai, China; 3 Key Laboratory of Sport Skill and Tactic Diagnosis and Analysis of General Administration of Sport of China, Shanghai, China; Government Law College, INDIA

## Abstract

This study investigates the relationship between referees’ physical fitness, their in-game performance, and team dynamics in the Chinese Super League (CSL). The sample comprised 173 matches from the 2021 CSL season, involving 30 referees (mean age 41 years). The Pearson product-moment correlation coefficient was used to analyze the correlation between referees’ physical fitness tests and their match performance indicator, as well as the referee’s game performance and the home and away teams’ game performance. Key findings include a significant positive correlation between referees’ RSA_VARIATION_ and the average speed during home possession (r =  0.16, p =  0.04). Additionally, RAS_BEST_ and RAS_AVERAGE_ were found to have small to moderate negative correlations with the referees’ total distance, average speed, and high-intensity activities during games (r =  -0.20 to -0.34, p < 0.001). Furthermore, significant positive correlations were observed between referees’ match performance metrics and the physical outputs of both home and away teams. These findings provide valuable insights for enhancing the understanding of factors influencing referee performance and can inform referee training, testing, and selection processes.

## 1. Introduction

Referees play a crucial role in football matches, as they are responsible for enforcing the rules and regulating players’ behaviors on the field [1]. Their decisions can directly impact the results of matches, especially in high-level professional games [2]. The competence of a referee is not solely based on their understanding of football rules but also heavily relies on their physical performance [3]. To observe the actions on the field and make prompt judgment calls, referees need to maintain appropriate distances from the players and constantly move around the pitch [4]. It has been reported that referees cover about 11 km per game with 10–15% of the overall distance at high running speed (>18 km/h), which is even more than players achieve on average [5]. This indicates that good physical fitness is crucial for referees’ performance. Physical fitness not only supports referees’ movement efficiency and accurate decision-making but also helps them maintain strong performance during high-intensity matches [6]. However, the performance of teams can significantly impact the physical demands placed on referees. For instance, high-intensity games or fast-paced play increase the physical burden on referees, requiring them to cover larger areas of the pitch and maintain higher levels of alertness [7]. During high-intensity moments, such as counterattacks, referees often need to sprint to keep up with the play, which can significantly elevate physical demands. These physical challenges not only test the referees’ fitness levels but also influence their officiating effectiveness, potentially impacting their ability to make accurate decisions and maintain control of the match [8]. By ensuring optimal fitness, referees may better manage the rigors of these demanding scenarios, thus enhancing decision-making accuracy and overall match management.

Research has identified the key fitness components most relevant for refereeing, including aerobic endurance, repeated sprint ability, agility, and muscular power [2]. To adequately evaluate referees’ capabilities, their performance should be assessed through fitness tests designed specifically for the sport. Therefore, the referee’s performance can be evaluated through a different battery of fitness tests to assess their football-related fitness levels [9]. Specifically, the 6 x 40 m sprints, 150 m interval run, and Yo-Yo test are commonly used to assess the physical fitness of football referees [10, 11]. In addition, the 5m, 10m, and 30m sprints, T-Test, Arrowhead, and Illinois Agility tests are also used for assessing the agility of football referees [12]. In prior research, there has been much discussion about the correlation between physical fitness tests and the match performance of referees. Specifically, Castillo et al. [13] investigated the correlation between specific match performance variables and the outcomes of fitness tests in male international football referees, and the results revealed that a high sprint and cardiovascular fitness level could be relevant to the performance of male referees during matches. Sánchez et al. [9] investigated the association between fitness level and the physical match demands of professional female football referees. The results revealed positive correlations between intermittent exercise fitness levels, repeated sprint ability performance, and match performance in female football referees.

Although previous studies have explored the relationship between referee fitness test results and match performance [9,13], very few studies have delved into the correlation between referee match performance and player match performance. The importance of this correlation lies in the fact that referees’ fitness not only affects their own performance but may also be influenced by the tactical and technical level of the teams on both sides of the game, which can further affect the fairness and outcome of the game [14]. Mascarenhas et al. [15] found that English referees in higher-level leagues covered greater total distances and more frequently entered critical areas in both halves compared to referees in lower leagues. This indicates referees adjust their positioning and physical exertion according to competition level, meaning the high-intensity running of players at different levels correlates positively with referee running intensity [4]. Additionally, teams with higher competitive skill levels often adopt more stable passing tactics, which may affect the referee`s positioning strategy [6]. This tactical difference not only affects the physical exertion of the referee but may also require the referee to make more precise judgments, further emphasizing the importance of correlation studies between referee game performance and team performance. By using a consistent passing technique, the referee can analyze the player`s passing route by reading the game and getting to the right position before the player passes the ball, thus reducing physical exertion. Specifically, from a technical and tactical standpoint, research suggests that referees tend to adopt closer decision-making distances during various player actions (including passing, dribbling, shooting, etc.) [4]. This is because decision-making accuracy diminishes as the distance between the referee and the action increases. Gilis et al. [16] found that assistant referees make more offside judgment errors during long passes. This demonstrates that different pass types affect the accuracy of assistant referees in judging player positions.

The physical fitness and performance of referees during matches are important, as highlighted by the various fitness tests used to evaluate referees [10, 11]. While previous studies have focused on the physical performance of soccer referees [9,13]. Limited research has explored how this performance correlates with the dynamic strategies and physical demands of both home and away teams. Therefore, the primary aim of this study is to assess the correlation between referees’ physical fitness (as measured in fitness tests) and their performance in officiating matches, as well as how these metrics relate to the performance of both home and away teams. By exploring this relationship, we aim to provide evidence-based insights that can enhance referee training, testing, and selection processes. Understanding these correlations will help in better preparing referees to meet the physical demands of their roles, thereby improving their ability to enforce game rules and maintain fairness in matches.

## 2. Materials and methods

### 2.1 Sample and data

Our data comes from CSL authorities. The Chinese Football Association (CFA) conducts annual fitness tests for referees officiating in the CSL and provides a comprehensive evaluation based on their performance during the season, aiming to ensure that referees will perform better in the following season. The fitness tests are administered by CSL’s professional staff, ensuring the testing process’s professionalism and the data’s credibility. The research is based on the annual physical fitness tests that the CFA conducts for referees. These tests are routine referee selection and evaluation activities, similar to examinations, not experimental studies. The participants are aware, as this is a standard part of their career progression. Thus, the data used in our study were obtained through standard evaluation and selection processes, not through experimental methods. This study included historical test data from selected 30 active referees with a mean age of 41. Concretely, all referees received identical training volume and intensity before testing. Each referee completed a repeated sprint ability test and an intermittent running test during testing. In the repeated sprint ability test, referees sprinted six times with one-minute recovery periods between sprints. The fastest sprint time was selected as the best performance (denoted Best, units s), while the average of the six trials was also recorded (denoted Average, units s). To assess fatigue resistance in the repeated sprint test, a coefficient of variation compared variability between trials (denoted variation, %). Coefficients above 15% indicated potentially abnormal variability requiring data exclusion [17, 18]. The total distance covered for the intermittent running test was recorded as the test result (denoted Distance, m). Finally, data were included on the on-field performance of the 30 CSL referees over the 2021 season and the on-field performance data of CSL teams. Data related to the referees’ physical performance and spatial information and the H/A team’s physical and technical performance were collected during 173 CSL matches from the 2021 season by Amisco using an optical tracking system whose accuracy, validity, and reliability have been confirmed by previous studies [19, 20].

### 2.2 Data processing and ethics statement

The data utilized in this study comprise routine physical fitness tests and match statistics of referees conducted by the CFA, rather than data acquired through experiments. Referees undergo these tests and officiate matches as part of their job requirements, akin to examinations, rather than being subjects of experimental research.

Prior to our acquisition and utilization of these data, all information has been anonymized, devoid of any personal identifying details.

The data does not originate from referees’ medical records or similar sources, thus exempting the necessity for obtaining informed consent waivers from an ethics committee.

Referees are informed about and consent to the utilization of their data for research purposes, as elucidated herein.

### 2.3 Procedure

One fitness test for the referees was the repeated sprint ability (RSA) test, which consisted of six 40-meter sprints with one minute of recovery between each sprint [21]. This sprint of 40 meters was monitored utilizing a system comprised of two pairs of photocells (Microgate, Bolzano, Italy) positioned at intervals of 0 and 40 meters, respectively [9,22]. The evaluation in this test pertains to the capacity for repeated high-intensity efforts and the assessment of physiological parameters, including but not limited to maximal oxygen uptake [23]. The variation (RSA_VARIATION_), the best time (RSA_BEST_), and the average time (RSA_AVERAGE_) were calculated. Additionally, the interval test assesses the referee’s ability to perform a series of high-speed 75-meter runs (maximum time allowed =  15 seconds) interspersed with 25-meter walking intervals (recovery time =  20 seconds). A total of 40 runs were conducted. The minimum speed requirement for the high-speed runs was 18 km/h. Referees completed 40 sets of 75-meter runs followed by 25-meter walks, totaling 4000 meters. After each run, the referee must enter the walking area before the command is given by the official test instructor (whistle). If a referee fails to place a foot inside the walking area on time, they will receive one warning from the test instructor. If a referee fails to place a foot inside the walking area on time a second time, they will be stopped by the test instructor and informed that they have failed the test.

From the initial dataset, we derived a total of 27 performance indicators (refer to Table 1), focusing on the referee’s physical performance and distance to the ball, as well as the physical and technical performance of the home and away teams across various game events. We measure all decision-making distance-related events in meters [4].

**Table 1:** Definitions of selected technical and physical performance-related parameters.

Referees’ physical fitness tests-related parameters: Operational Definition
**RSA**_**BEST**_: The shortest time in the repeated sprint ability test. **RSA**_**AVERAGE**_: The average time of the repeated sprint ability test. **RSA**_**VARIATION**_: The coefficient of variation was used to compare the magnitude of variation, i.e., the fluctuation between test scores, and if the coefficient of variation was greater than 15%, it was considered for exclusion. **Interval test distance (m)**: The distance taken or run during the test will be calculated as a composite of distances, indicated by Distance.
**Referees Performance-Related Parameters: Operational Definition**
**Total distance covered (m)**: Distance covered in a match. **Average speed (km/h)**: Average speed in the match. **Average speed during home possession (km/h)**: Average speed when home team has the ball possession. **Average speed during away possession (km/h)**: Average speed when away team has the ball possession. **Number of high-intensity running**: Number of high-intensity-running in a match **Distance high-intensity running (m)**: Distance covered at the speed over 19 km/h in a match. **Decision making distance**: The spatial distance within which a referee needs to make critical decisions during a match.
**H/A Team’s physical and technical performance-related parameters: Operational Definition**
**Pass**: An intentional ball played from one player to his teammate. **Possession (%)**: The percentage of time during a match that a particular team has possession of the ball. **Successful pass (%)**: Successful passes as a proportion of total passes. **Forward pass**: Intentional played balls from one player to another who is located in opponent’s half of pitch. **Successful forward pass (%)**: Successful forward passes as a proportion of total forward passes. **Average length of pass**: The average distance of passes completed by a team during a match. **Passes per shot**: The total number of passes divided by the total number of shots. **Gain of possession**: The action of gaining possession from an opposition player who is in possession of the ball. **Loss of possession**: The moment when a player who had control of the ball loses it to an opponent, either through a mistake or due to the opponent’s skillful intervention. **Ball retention (%)**: Ball retention percentage refers to the percentage of time a team to keep possession of the ball during a match. **Distance (m)**: Distance covered in a match. **Average speed (km/h)**: Average speed in the match. **Average speed during home possession (km/h)**: Average speed when home team has the ball possession. **Average speed during away possession (km/h)**: Average speed when away team has the ball possession. **Number of high-intensity running**: Number of high-intensity-running in a match by all the players of a team. **Distance high-intensity running (m)**: Distance covered at the speed over 19 km/h in a match by all the players of a team.

### 2.4 Statistical analysis

The Pearson product-moment correlation coefficient was used to assess the strength and direction of the linear relationship between continuous variables, such as referees’ fitness test results and match performance. This method was chosen due to its appropriateness in analyzing linear relationships when normality is assumed, as confirmed by the Kolmogorov–Smirnov test. Pearson’s correlation is widely used in sports science research to quantify the strength of linear relationships between continuous variables, making it suitable for this dataset.

The descriptive statistics were presented as mean ±  standard deviations. Firstly, a Kolmogorov–Smirnov test was used to test the normality of the data. Secondly, the relationship between referees’ physical fitness tests and their match performance indicator was investigated using the Pearson product-moment correlation coefficient. Finally, the Pearson product-moment correlation coefficient was used to analyze the correlation between the referee’s game performance and the home and away teams’ game performance (physical and technical indicators).

Prior to analysis, the data was examined to ensure it met the assumptions of normality, linearity, and homoscedasticity as recommended by Pallant [24]. To handle potential outliers, standardized residuals greater than ± 3 standard deviations from the mean were flagged for further inspection. Outliers were removed if they were identified as errors or unrepresentative cases.

The following scales were used to classify the correlation strength: very small, 0–0.1; small, 0.1–0.3; moderate, 0.3–0.5; large, 0.5–0.7; very large, 0.7–0.9; 0.9–1, nearly perfect; 1, perfect [25]. A significance level of *p* <  0.05 was applied to determine statistical significance, as it is widely accepted in sports science research to strike a balance between sensitivity and risk of Type I errors [26]. Statistical analyses were performed using SPSS statistics software version 26 (IBM, Armonk, NY, USA).

## 3. Result

### 3.1 Referees’ physical fitness tests and their match performance

**Fig 1:**
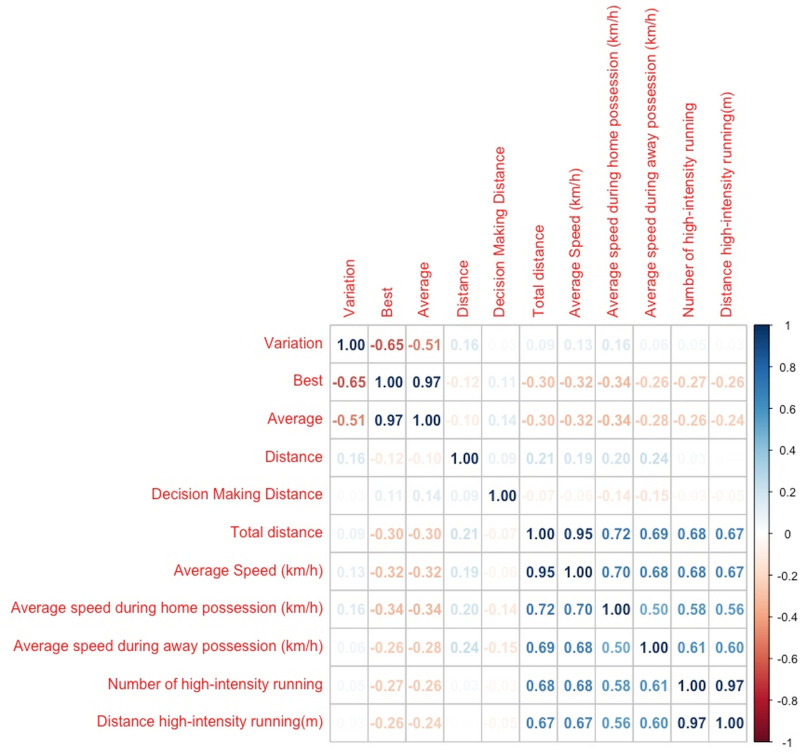
The correlation between referees’ physical fitness tests and their physical performance in matches.

The correlations between referees’ fitness test performance and match performance are shown in Fig 1 and Table 2. The referees’ RSA_VARIATION_ was positively correlated with average speed during home possession (r =  0.16, *p* =  0.04). However, the referees’ RAS_BEST_ and RAS_AVERAGE_ showed a small to moderate negative correlation with the total distance, average speed, average speed during home possession, average speed during away possession, number of high-intensity running, and distance of high-intensity running (r =  -0.2 to -0.34, *p* <  0.001, respectively). Finally, the referee’s interval test distance demonstrated a positive correlation with the total distance, average speed, average speed during home possession, and average speed during away possession (r =  0.19–0.24, *p* <  0.01, respectively).

**Table 2:** The key correlations between referees’ test performance and match performance.

	RSA_Variation_	RSA_Best_	RSA_Average_	Distance	DMD	RTD	RAS	ASDAP	ASDAP	NHIR	DHIR
**RSA**_**Variation**_	**1**										
**RSA**_**Best**_	**-.649** ^**^	**1**									
**RSA**_**Average**_	**-.520** ^**^	**.972** ^**^	**1**								
**Distance**	**.164** ^*^	**-0.115**	**-0.094**	**1**							
**DMD**	**0.007**	**0.095**	**0.134**	**0.034**	**1**						
**RTD**	**0.085**	**-.302** ^**^	**-.300** ^**^	**.213** ^**^	**-0.075**	**1**					
**RAS**	**0.128**	**-.321** ^**^	**-.322** ^**^	**.191** ^*^	**-0.059**	**.946** ^**^	**1**				
**ASDAP**	**.157** ^*^	**-.339** ^**^	**-.340** ^**^	**.202** ^**^	**-0.137**	**.719** ^**^	**.704** ^**^	**1**			
**ASDAP**	**0.064**	**-.258** ^**^	**-.279** ^**^	**.236** ^**^	**-0.145**	**.689** ^**^	**.677** ^**^	**.505** ^**^	**1**		
**NHIR**	**0.047**	**-.269** ^**^	**-.260** ^**^	**0.031**	**-0.034**	**.677** ^**^	**.679** ^**^	**.581** ^**^	**.605** ^**^	**1**	
**DHIR**	**0.03**	**-.256** ^**^	**-.244** ^**^	**0.003**	**-0.048**	**.669** ^**^	**.666** ^**^	**.564** ^**^	**.597** ^**^	**.970** ^**^	**1**

**DMD** represents the decision-making distance (m), **RTD** refers to the total distance covered (m), **RAS** is the average speed (km/h), **ASDHP** is the average speed during home possession (km/h), **ASDAP** is the average speed during away possession (km/h), **NHIR** stands for the number of high-intensity running events, and **DHIR** refers to the distance covered during high-intensity running (m). *p < 0.05; **p < 0.01.

### 3.2 Referees’ match performance and home team’s performance

**Fig 2:**
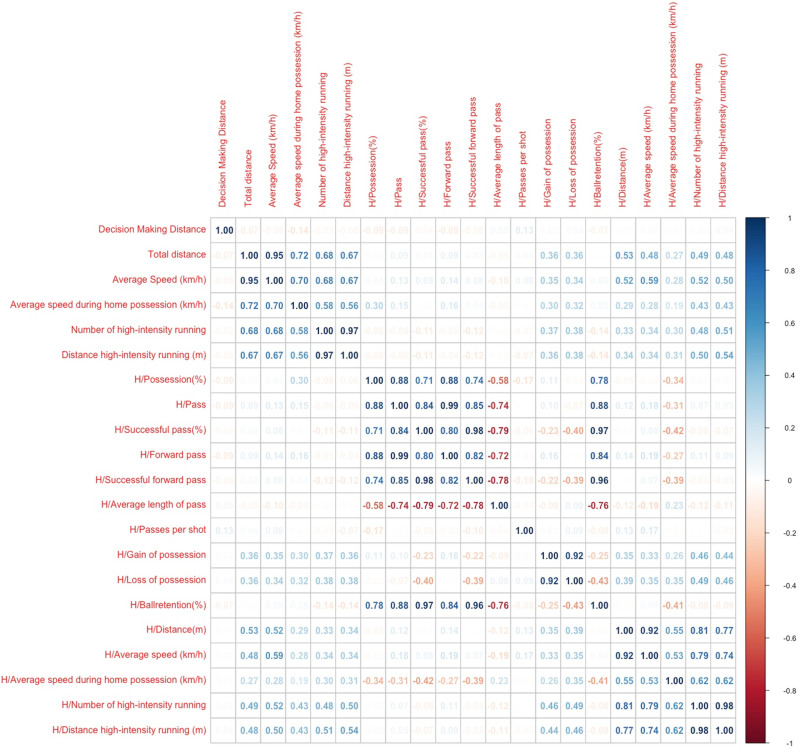
The correlation between referees’ physical performance and home team’s match performance.

Correlations between referees’ match performance and the home team’s performance are highlighted in Fig 2. The referee’s total running distance and average speed were positively correlated with the home team’s gain and loss of possession, average speed, total distance, average speed during home possession, number of high-intensity running, and distance of high-intensity running (r =  0.34-0.59, *p* <  0.05, respectively). Furthermore, small to moderate positive correlations were found between the referee’s average speed during home possession and the home team’s total distance, forward pass, gain and loss of possession, average speed, number of high-intensity running, and distance of high-intensity running (r =  0.16-4.3, *p* <  0.05, respectively). Finally, the referee’s number of high-intensity running and distance of high-intensity running were positively correlated with the home team’s gain and loss of possession, average speed, total distance, average speed during home possession, number of high-intensity running, and distance of high-intensity running (r =  0.32–0.54, *p* <  0.05, respectively).

### 3.3 Referees’ match performance and away team’s performance

**Fig 3:**
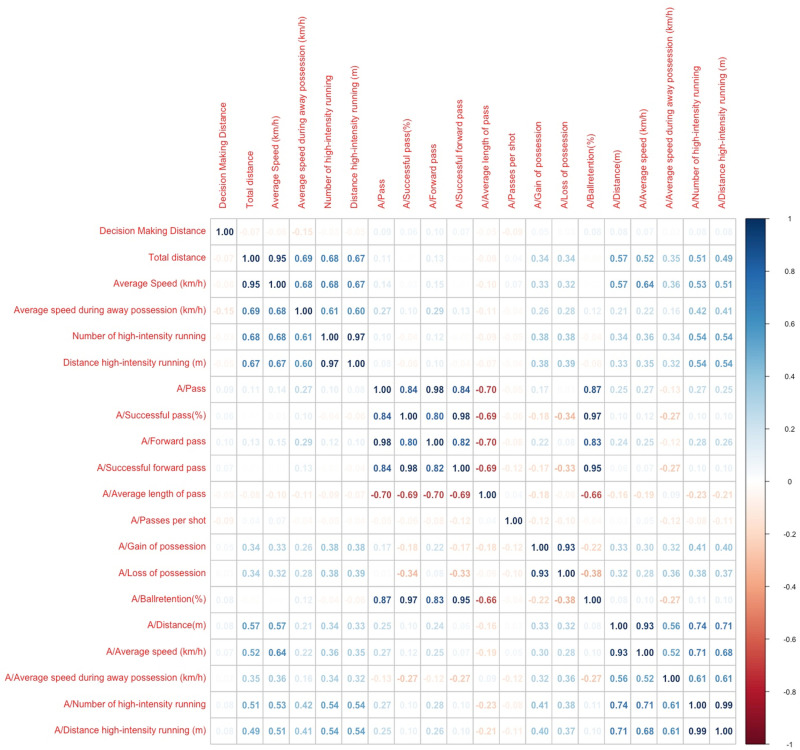
The correlation between referees’ physical performance and away team’s match performance.

Correlations between referees’ match performance and the away team’s performance are presented in Fig 3. Specifically, the referees’ total distance and average speed showed a moderate to large correlation with the away team’s gain and loss of possession, total distance, average speed, average speed during away possession, number of high-intensity running, and distance of high-intensity running (r =  0.32-0.65, *p* <  0.05, respectively). Moreover, the results demonstrated that the average speed during away team possession of referees displayed a small to moderate positive correlation with the away team’s pass, forward pass, gain and loss of possession, total distance, average speed, average speed during away possession, number of high-intensity running, and distance of high-intensity running (r =  0.16-0.42, *p* <  0.05, respectively). Finally, the number of high-intensity running and the distance of high-intensity running performed by referees demonstrated a positive correlation with the away team’s gain and loss of possession, average speed, total distance, average speed during away possession, number of high-intensity running, and distance of high-intensity running (r =  0.32-0.54, *p* <  0.05, respectively).

## 4. Discussion

The aim of this study was twofold: firstly, to examine the connection between referees’ performance in fitness tests and their performance during matches; secondly, to investigate the potential correlation between referees’ match performance and the performance of both home and away teams. The key finding revealed a significant association between referees’ performance on fitness tests and the physical demands placed on them during matches. Thus, assessing football referees’ physical fitness through standardized tests is crucial for ascertaining their capacity to effectively officiate matches [6].

In soccer matches, especially during fast attacks or counterattacks, teams often place demands on referees to repeatedly sprint and react swiftly [6]. As a result, referees possessing superior RSA can effectively keep pace with the game’s rhythm, maintain a precise understanding of the game situation, and render more accurate and impartial judgments [27, 28]. Referees’ RSA_VARIATION_ represented stability across the six repeated sprints, with better RSA_VARIATION_ indicating relatively superior anaerobic recovery ability [18]. In this scenario, there was only a small positive correlation between referees’ RSA_VARIATION_ and their average speed during home possession, implying that referees frequently need to keep pace with the home team’s speed during possession. This finding suggests specific requirements for referees to engage in repeated sprinting during home team possession, which aligns with the findings of a prior study [6]. Additionally, referees’ RSA_BEST_ and RSA_AVERAGE_ in fitness tests demonstrated small to moderately negative correlations with all their physical parameters during the game. This suggests that referees who perform better in fitness tests tend to exhibit relatively higher running output during matches. These findings imply that while superior repeated sprint ability (RSA) contributes to higher physical outputs, other factors, such as game-reading ability and experience, may play significant roles in moderating the relationship between fitness test outcomes and in-game performance. For instance, referees with strong RSA may be better equipped to sustain high-intensity efforts throughout a match, facilitating timely positioning and reducing decision-making distances. However, this reliance on physical capacity underscores the need for balanced training programs that integrate both physical conditioning and cognitive development to improve match management. To improve referee training and evaluation, programs should combine RSA and endurance training with cognitive drills to enhance decision-making under fatigue. Including game-specific metrics, such as decision accuracy, can ensure referees are well-prepared for the physical and mental challenges of modern football. Furthermore, there was a positive correlation between the referees’ interval test distance and various game-related metrics, including total distance, average speed, average speed during home possession, and average speed during away possession. This finding suggests that referees exhibited superior endurance and physical adaptability in the interval test, which facilitates maintaining high activity and responding swiftly to varying game situations during matches [29]. The dynamicity and ever-changing situations in soccer require referees to move accurately and rapidly to penalty positions for impartial decisions [30]. Thus, the positive correlations between interval test distance and several key in-game parameters may indicate referees’ robust endurance helps them adapt better to game rhythm and intensity.

Previous studies have established a connection between the physical performance of soccer referees and players during a match [1,29,31]. However, the correlation between referees’ performance and the performance of home and away teams during matches has been poorly studied. In this study, it was found that the referee’s total running distance and average speed were positively correlated with the home team’s average speed, total distance, average speed during home possession, number of high-intensity running, and distance of high-intensity running. This suggests that referees may unconsciously favor the home team by maintaining closer proximity and better positioning to them during matches, which may reflect to some degree the home advantage caused by referee bias [32–34]. Similar trends have been noted in other major leagues such as the Premier League, Bundesliga, La Liga, and the Champions League [35–37]. Furthermore, previous studies have indicated that referees may employ varying physical strategies when officiating different types of matches and teams [30,31]. Similarly, our study found that the referee’s average speed during home possession was positively related to several indicators of the home team. This result may be attributable to the distinct match strategies employed by the home and away teams. Specifically, Gómez et al. [38] suggested that home teams often prefer possession-based styles of play, while away teams tend to adopt defensive or direct approaches. This distinction in playing styles can result in longer ball possession times for the home team, providing referees with increased opportunities to engage in more high-intensity physical performances. Moreover, Weston et al. [29] found that the physical performance of soccer referees during a match was positively correlated with the high-intensity running distance of players. Similarly, our results showed that the referee’s number of high-intensity running and distance of high-intensity running were positively correlated with the home team’s average speed, total distance, average speed during home possession, number of high-intensity running, and distance of high-intensity running. This may demonstrate that the referee’s high-intensity running activity during the game is closely related to the home team’s game characteristics, suggesting that the referee’s physical and motor activity is critical to game observation and decision-making. Furthermore, high-intensity running can reduce the decision-making distance of high-level soccer referees during a match [2,29], which may help them get closer to match events for better observation and analysis [39].

The current findings demonstrate significant positive correlations between referees’ match performance parameters and away teams’ match performance metrics. Specifically, referees’ total distance and average speed showed moderate to large correlations with multiple indicators of away teams’ physical performance. This aligns with previous research [2], which reported that greater distance covered by referees was associated with increased physical demands placed on teams during matches. Moreover, our observations indicated that the connection between referees’ decision-making distance and away teams’ performance indicators is relatively weak. This suggests that referees’ decision-making distance may depend more on individual judgment and professional skills than significant external influences. Jiang et al. [4] also stated the lack of interaction between the physical activity indicators and the decision-making distances of referees in different types of matches and halves. While the average speed of referees during the away team’s possession only showed small positive correlations with most of the away team’s performance indicators, it did demonstrate moderate positive correlations with the away team’s high-intensity running frequency and distance. One potential explanation is that higher team speed implicitly raises the tempo and intensity of the match, which could prompt the referees to increase average speed to catch up with the match pace and make timely decisions. This explanation is supported by the findings of Mallo et al. [40], which demonstrated that higher referee speed correlates with increased match intensity. Additionally, the high-intensity running frequency and distance covered by referees were moderately correlated with the away team’s gain and loss of possession, total distance, average speed, and average speed during possession. Referees’ high-intensity running indicators also showed a large positive correlation with the away team’s number of high-intensity running and distance of high-intensity running. This demonstrated that the performance of referees on the pitch correlates with the intensity of the away team’s high-intensity physical activities. Concurrently, the increase in high-intensity running by players during the match imposes higher physical fitness demands on referees [29]. When the match intensifies, referees require superior physical conditioning to adapt to the pace of the game to make more accurate judgments, thereby upholding the impartiality of the match [29,41]. Therefore, the high-intensity activities of referees are closely associated with the intensity and trends of the match, reminding referees to sustain appropriate activity levels and reaction speeds under high-pressure conditions [2].

## 5. Conclusion

This study systematically identified significant correlations between referees’ in-game performance metrics, fitness test results, and team performance in the CSL. The findings demonstrated that referees’ RSA_VARIATION_ was positively correlated with the home team’s average speed during possession. In contrast, RAS_BEST_ and RAS_AVERAGE_ exhibited small to moderate negative correlations with referees’ total distance, average speed, and high-intensity activities during matches. Additionally, referees’ total running distance and average speed were positively associated with key performance indicators of both home and away teams, including average speed, distance covered, and high-intensity running.

These findings highlight the need to align referee training programs with the physical demands observed during matches. By integrating key correlations into training, referees can better prepare for different match scenarios. Tailoring programs to improve critical physical attributes like agility and sprint capacity could enhance positioning and decision-making in high-intensity moments. The results also suggest that referee selection could benefit from incorporating both match performance data and fitness test results. This dual approach would enable selection committees to make more informed choices, potentially improving officiating quality. Finally, understanding the link between referee performance and team dynamics can guide referee assignments, ensuring the right officials manage specific match types, thus reducing controversies and improving game flow.

In summation, this study identified a discernible relationship between referees’ physical capabilities, match performance, and the movement dynamics of teams during games. Referee training programs should incorporate fitness tests focused on sprinting and endurance to align with the demands of the game. Additionally, referee selection committees could utilize these findings to ensure that referees possess the requisite physical preparation for high-intensity matches.

## 6. Limitations

While this study provides valuable insights into the relationship between referees’ physical fitness and match performance, several limitations should be acknowledged. First, the study did not account for external variables that could have influenced the results. For instance, meteorological conditions, such as extreme temperatures or humidity, may affect both referee and player performances. Future research could consider controlling for weather conditions to isolate the relationships observed better. Additionally, the significance of individual matches, such as high-stakes finals versus regular-season games, might also impact the physical and psychological demands placed on referees, potentially influencing their performance. Lastly, the presence (or absence) of spectators, as highlighted by studies during the COVID-19 pandemic, has been shown to affect player behavior and referee decision-making, potentially contributing to home advantage or referee bias. These factors were not measured in this study but could be critical in fully understanding the dynamics between referees’ fitness and their match-day performance. Future studies should explore these variables to provide a more comprehensive understanding of referee performance in various match environments.

## 7. Practical applications

The findings of this study suggest several practical applications for optimizing referee training and selection processes. The observed positive correlations between referees’ physical performance and match demands underscore the need for tailored training programs that target specific fitness components essential for effective officiating.

**Endurance training** Given the significant association between referees’ interval test performance and in-game metrics, it is crucial to incorporate endurance-focused training. Referees should engage in interval running sessions that simulate match conditions, such as high-speed 75-meter runs interspersed with walking or low-intensity recovery intervals. This approach will enable referees to sustain high levels of physical activity and respond efficiently to the fluctuating demands of a match over extended durations.

**Agility and sprint training** The findings indicate that referees who perform well in repeated sprint ability tests tend to have higher running outputs during matches. Therefore, agility drills, such as the T-Test and Illinois Agility Test, along with repeated sprint exercises (e.g., 6x40-meter sprints with one-minute recovery periods), should form a key part of referee training programs. These exercises will enhance referees’ ability to change direction rapidly and maintain optimal positioning, which is critical for accurate decision-making in high-intensity match scenarios.

**High-intensity interval training (HIIT)** The correlation between referees’ high-intensity running metrics and the physical performance of both home and away teams suggests that HIIT could improve their capacity to handle fast-paced, dynamic matches. Training sessions should include sprints at speeds exceeding 19 km/h, followed by short recovery periods, to replicate the physical demands of officiating quick transitions and counter-attacks during a match.
